# Overexpression of leucocyte common antigen (LAR) P-subunit in thyroid carcinomas

**DOI:** 10.1038/sj.bjc.6600876

**Published:** 2003-04-15

**Authors:** N Konishi, K Tsujikawa, H Yamamoto, E Ishida, M Nakamura, K Shimada, K Yane, H Yamashita, S Noguchi

**Affiliations:** 1 Department of Pathology, Nara Medical University, 840 Shijo-cho, Kashihara, Nara 634-8521, Japan; 2 Department of Immunology, Graduate School of Pharmaceutical Science, Osaka University, Yamadaoka 1-6, Suita, Osaka 565-0871, Japan; 3 Department of Otolaryngology, Nara Medical University, 840 Shijo-cho, Kashihara, Nara 634-8521, Japan; 4 Noguchi Thyroid Clinic and Hospital Foundation, Noguchinakamachi 6-33, Beppu , Oita 874-0932, Japan

**Keywords:** LAR, P-subunit, PTP, thyroid carcinoma, immunohistochemistry

## Abstract

Protein tyrosine phosphatase (PTPase) dephosphorylation and protein tyrosine kinase (PTKs) phosphorylation of key signal transduction proteins may be regulated by extracellular signals, making PTPases important in the regulation of cell proliferation. Leucocyte common antigen (LAR), a receptor-like PTPase, consists of E-subunit, containing the cell adhesion molecule-like receptor region, and P-subunit specific for a short segment of the extracellular region, the transmembrane peptide, and two cytoplasmic PTPase domains. We produced a monoclonal antibody against the LAR P-subunit for immunohistochemical screening of LAR expression in normal and tumourous tissues. Gliomas and gastric, colorectal, lung, breast and prostate cancers showed weak and relatively infrequent expression. Intense and diffuse expression, however, was detected in 95% (227 out of 239) of thyroid carcinomas, but only 12% (22 out of 128) of adenomas and no cases of benign thyroid disease were immunopositive. In contrast to broad staining in carcinomas, LAR expression in thyroid adenomas was often found in small focal or locally invasive areas. Western blot analysis similarly detected LAR P-subunit protein in thyroid carcinomas, but not in normal tissues. We believe this to be the first demonstration of LAR overexpression in thyroid carcinoma and may help to elucidate the role of PTPases in the development of malignancy.

Protein tyrosine phosphatases (PTPases) are important regulators in the reversible tyrosine phosphorylation of cellular protein via their interactions with protein tyrosine kinases (PTKs). Many oncogenes encode PTKs that participate in a large set of cellular events, such as migration, proliferation, differentiation and transformation, by regulating tyrosine phosphorylation of cellular proteins (Chernoff, 1999). The observation that PTPase can counteract PTK has led to the hypothesis that certain PTP genes might behave as tumour suppressor genes (Hunter, 1989). The fact that expression of certain PTPases is upregulated in hepatoma cells and in breast and glial tumours lends weight to the hypothesis and has identified some specific PTPases as candidates that might influence oncogenic transformation and tumour growth (Zhai *et al*, 1993; Wiener *et al*, 1994; Elson and Leder, 1995; Li *et al*, 1996; Norman *et al*, 1998).

To date, PTPases are structurally divided into intracellular PTPases and transmembrane receptor-like PTPases (Walton and Dixon, 1993; Neel and Tonks, 1997). Although intracellular PTPases possess only one PTPase domain, receptor-like PTPases have two cytoplasmic PTPase domains repeated in tandem. Among the receptor-like PTPases, leucocyte common antigen (LAR) consists of two noncovalently bound subunits, the extracellular (E) subunit and the phosphatase (P) subunit. The 150-kDa E-subunit is composed of three Ig domains and eight Fn III domains, whereas the 85-kDa P-subunit has a short extracellular domain, a transmembrane domain and two tandemly repeated PTPase domains, 1 and 2 (Streuli *et al*, 1992, 1998; Tsujikawa *et al*, 2001). Previous investigations suggest that domain 2 is structurally very similar to domain 1 and that its function may be to regulate the catalytic activity or specificity of domain 1 (Krueger *et al*, 1990; Streuli *et al*, 1990; Wang and Pallen, 1991; Nam *et al*, 1999). LAR has been reported to play a regulatory role in insulin signalling via the extracellular and two PTPase domains of the P-subunit (Hashimoto *et al*, 1992; Zhang *et al*, 1994; Serra *et al*, 1995; Tsujikawa *et al*, 2001). Analysis of LAR mutant proteins suggests that domain 1 is responsible for insulin receptor dephosphorylation, while domain 2 functions in recognition of the phosphorylated insulin receptor.

Although increased expression of LAR has been reported in breast cancer and in pheochromocytoma (Yang *et al*, 1999, 2000), the antibody used in these studies was raised against the E-subunit, rather than the P-subunit. To address the possibility that the LAR P-subunit might exert PTP-influencing oncogenic activity, we immunohistochemically screened the distribution of the P-subunit in both normal tissues and in various human tumours, subsequently concentrating on a large series of thyroid tumours in which overexpression of LAR P-subunit apparently distinguished malignant from benign disease.

## MATERIALS AND METHODS

### Tissue specimens

All normal tissues examined were obtained from three autopsy cases. Tumour samples were obtained from surgery cases and included 13 gliomas, 10 meningiomas, 21 thyroid carcinomas, 16 gastric cancers, 26 colorectal cancers, 20 lung cancers, 20 breast cancers, eight hepatocellular carcinomas, 21 renal cell carcinomas and 32 prostate cancers. No chemotherapy was instituted before tumour excision. We also obtained additional thyroid tumours and samples of benign thyroid diseases obtained from surgical cases, which included 211 papillary carcinomas, 28 follicular carcinomas, two anaplastic carcinomas, two medullary carcinomas, 128 follicular adenomas, five nodular goiters, seven specimens from patients suffering from Basedow's disease (diffuse toxic goiters) and six cases of Hashimoto's thyroiditis. Tissue specimens were fixed in 10% neutral-buffered formalin and embedded in paraffin, and contiguous sections cut at 4 *μ*m for H&E staining and immunohistochemical analyses. Additional portions of thyroid tumours and samples of benign disease were frozen at −80°C for later Western blot analysis.

### Preparation of anti-LAR P-subunit monoclonal antibody

Glutathione-S-transferase-LAR fusion protein (GST–LAR) was employed as an immunogen. *Escherichia coli* AD202 was transformed with the pGEX-2T expression vector (Amersham Bioscience Corp., Piscataway, NJ, USA), which was incorporated to its *Bam*HI/*Eco*RI site with cDNA corresponding to 607 amino acids spanning from the end of the transmembrane region of the LAR P-subunit (amino-acid residues 1275–1881) through the entire cytoplasmic region. BALB/c mice were immunised four times at approximately 2-week intervals with 50 *μ*g of the fusion proteins. At 4 days after the last immunisation, splenocytes from mice with elevated antibody titres were fused with cells from the BALB/c-derived myeloma cell stain NS1 and the resulting hybridomas were cloned using the ClonaCell™-HY Hybridoma Cloning Kit (StemCell Technologies, Inc., Vancouver, Canada). We used ELISA to screen the culture supernatant of the cloned hybridoma using the recombinant GST–LAR cytoplasmic domain fusion proteins. The specificity of the anti-LAR P-subunit monoclonal antibody, which we designated YU1, was further confirmed by Western blotting on cell lysates from LAR-transfected COS-7 cells (Tsujikawa *et al*, 2001).

### Immunoblotting

Three representative frozen samples were divided into cancerous area and the corresponding normal tissue as positive and negative controls. The 300 *μ*g of proteins obtained from cell lysates of frozen samples were transferred from SDS–polyacrylamide gels to nitrocellulose membranes (Schleicher & Schuell Bio Science, Keene, NH, USA) and endogenous enzyme activity blocked with 3% bovine serum albumin (BSA) TBS-T (20 mM Tris-HCl pH 8.0, 137 mM NaCl and 0.1% Tween 20). The membranes were then incubated with 0.2 *μ*g ml^−1^ of the anti-LAR P-subunit monoclonal antibody at room temperature for 1 h, then washed three times with TBS-T. To detect anti-body binding, the membranes were exposed to horseradish peroxidase (HRP)-conjugated anti-rabbit IgG (Santa Cruz Biotech, Inc., Santa Cruz, CA, USA) diluted in TBS-T and incubated at room temperature for 1 h. After three washings in TBS-T, bound HRP conjugates were visualised with an enhanced chemiluminescent reagent (Wako Pure Chemical, Industries, Ltd, Osaka, Japan).

### Immunohistochemistry

Unstained formalin-fixed tissue sections were probed for antibody reactivity using the streptavidin–biotin (SAB) method as follows: sections were deparaffinised and endogenous peroxidase was blocked using 0.3% hydrogen peroxidase in methanol followed by heating for 5 min in 10 mmol l^−1^ of sodium citrate buffer (pH 6.0) in a pressure cooker, then rinsed with phosphate-buffered saline (PBS) and incubated for 20 min with dilute normal rabbit serum. Sections were incubated again at room temperature for 90 min with our anti-LAR P-subunit monoclonal antibody at a concentration of 0.1 *μ*g ml^−1^. Tissue sections were then exposed to biotin-labelled rabbit anti-mouse IgG for 30 min, followed by the binding reactions using a Histofine SAB-PO kit (Nichirei, Tokyo, Japan). Copious washing with PBS between each step was essential. The peroxidase reactions were visualised by exposure to a solution of 3, 3′-diaminobenzidine tetrahydrochloride in 0.01% hydrogen peroxide and counterstained with haematoxylin for microscopic evaluation. Identically treated specimens of thyroid cancer and the normal thyroid, which were immunopositive or negative for Western blotting, were used for the positive and negative controls.

In evaluating the immunostaining, we selected three areas within each stained lesion and counted the number of immunoreactive cells in fields of at least 200 tumour cells at a magnification of × 200. The relative degree of immunostaining was recorded as (−) when less than 20% of cells were positive, as (+) when 20–50% of cells were positive and as (++) when over 50% of cells were positive. Appropriate positive and negative controls were also included and evaluated in the same manner. Statistical analyses were carried out using Fisher's exact test supplemented by the Bonferroni procedure, a method that decreases the *α* level by dividing *α* by the number of comparisons made. Results were considered significant at *P*<0.05.

## RESULTS

**Figure 1:**
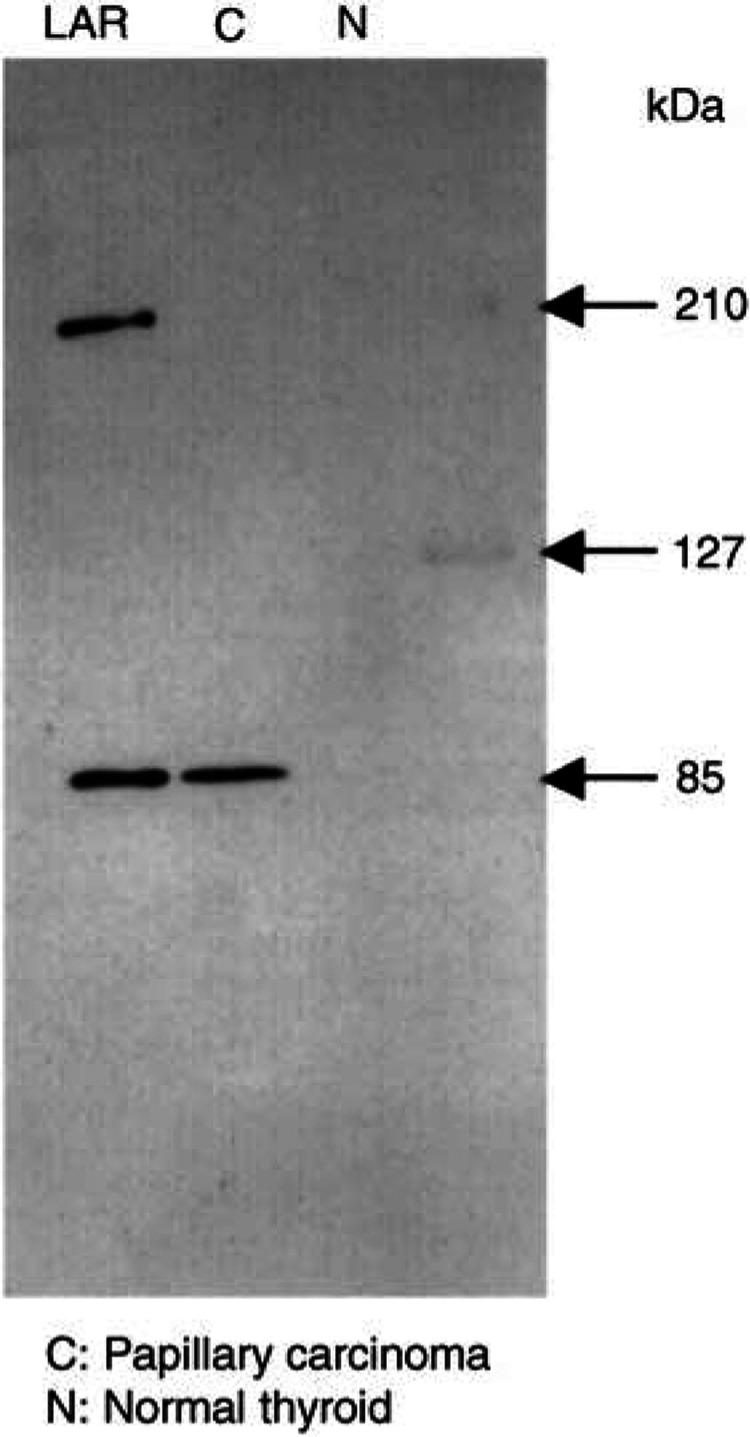
Western blot analysis of YU1 expression in thyroid carcinoma and in corresponding normal tissues. A weight of 300 *μ*g of each protein was loaded on polyacrylamide gels and transferred to nitrocellulose membrane. Leucocyte common antigen precursor and P-subunit are shown in the left lane at 200 and 85 kDa, respectively.

Leucocyte common antigen is synthesised as an approximately 200-kDa precursor protein that is cleaved by an endogenous protease into two subunits, the 150-kDa E-subunit and the 85-kDa P-subunit (Tsujikawa *et al*, 2001). The anti-LAR P-subunit monoclonal antibody, designated YU1, detected 200- and 85-kDa protein bands, which correspond precisely to the precursor protein and its P-subunit, respectively (Figure 1

).

To identify the distribution of the LAR P-subunit in normal tissues, we screened the expression in almost all organs of the body. Although less than 20% of cells were positive, LAR P-subunit expression was detected in heart (three out of three), the hepatocytes of the liver (three out of three), renal tubular epithelium (three out of three), the exocrine cells of the pancreas (three out of three), stomach, small and large intestine (three out of three), the ganglia (two out of two) and in testicular tissue, including the seminiferous tubules and Leydig's cells (two out of three). Normal thyroid glands were very weakly positive in two of three cases. The only strongly positive cells were the bronchial cells of the lung (three out of three, data not shown).

Table 1

**Table 1:** Expression of LAR P-subunit (YU1) in human tumours

		**Immunoreactivity**			
**Tumour**	**No. of cases**	**(−)**	**(+)**	**(++)**	**Percentage of positivity**
Brain tumour:					
Glioma	13	12	1	0	7.7
Meningioma	10	10	0	0	0
Thyroid carcinoma	21	0	8	13	100
Gastric cancer	16	12	1	0	6.3
Colorectal cancer	26	16	10	0	38.5
Lung cancer	20	18	2	0	10.0
Breast cancer	20	17	3	0	15.0
Hepatocelluar carcinoma	8	8	0	0	0
Renal cell carcinoma	21	21	0	0	0
Prostate cancer	32	30	2	0	6.3

**Figure 2:**
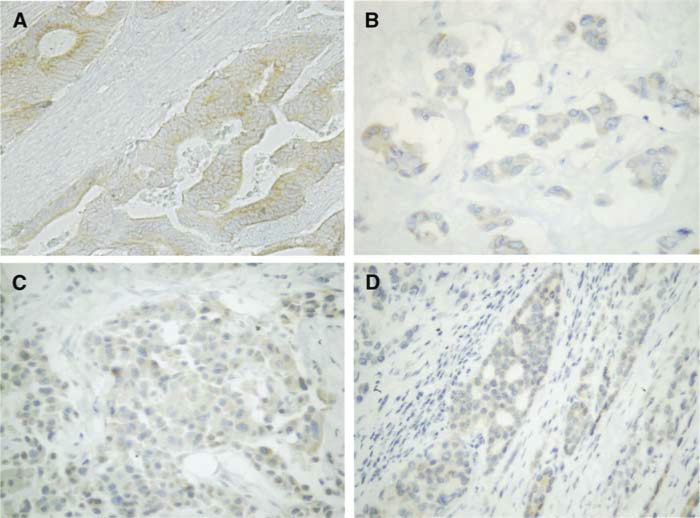
Immunohistochemical expression of LAR P-subunit (YU1) in human tumours. All cases were judged as (+). (× 200) (**A**) colorectal cancer; (**B**) poorly differentiated lung adenocarcinoma; (**C**) invasive ductal breast carcinoma; (**D**) prostate cancer, metastatic site.

Immunoreactivity was expressed as: (−) <20% of cells immunopositive; (+) 20–50% of cells immunopositive; (++) >50% of cells immunopositive.

illustrates the immunohistochemical detection of YU1 expression in various types of human tumours. All thyroid cancers examined were positive, with the 13 cases of papillary carcinomas showing a signal significantly stronger (*P*<0.001) than that seen in any other tumour type. Although the normal gastrointestinal mucosal glands exhibited very weak reactivity to YU1, gastric and colorectal cancers showed a diffuse staining pattern (Figure 2A

). Two of 20 cases of lung cancer, adenocarcinomas (10%) were focally positive for YU1 (Figure 2B). Each of the three cases of YU1-positive breast cancer (three of 20 cases, or 15%) was histologically invasive ductal carcinoma (Figure 2C). Only two of 32 prostate cancers (6.25%) were immunopositive; one case histologically appeared to be potentially metastatic (Figure 2D) and the other was immunoreactive only in frankly invasive areas.

Owing to the striking strong immunoreactivity of the thyroid cancers, we turned our attention to this apparently specific expression of LAR P-subunit in thyroid lesions, examining a large and varied series of thyroid tumours and diseases. Table 2

**Table 2:** Expression of LAR P-subunit (YU1) in thyroid tumours and diseases

		**Immunoreactivity**						
**Thyroid tumour**	**No. of cases**	**(−)**	**(+)**	**(++)**	**Percentage of positivity**			
Papillary carcinoma	211	9	115	87	95.7
Follicular carcinoma	28	3	19	6	89.3
Anaplastic carcinoma	2	2	0	0	0
Medullary carcinoma	2	2	0	0	0
Follicular adenoma	128	106	21	1	17.2
Nodular goiter	5	5	0	0	0
Basedow's disease	7	7	0	0	0
Hashimoto's thyroiditis	6	6	0	0	0

**Figure 3:**
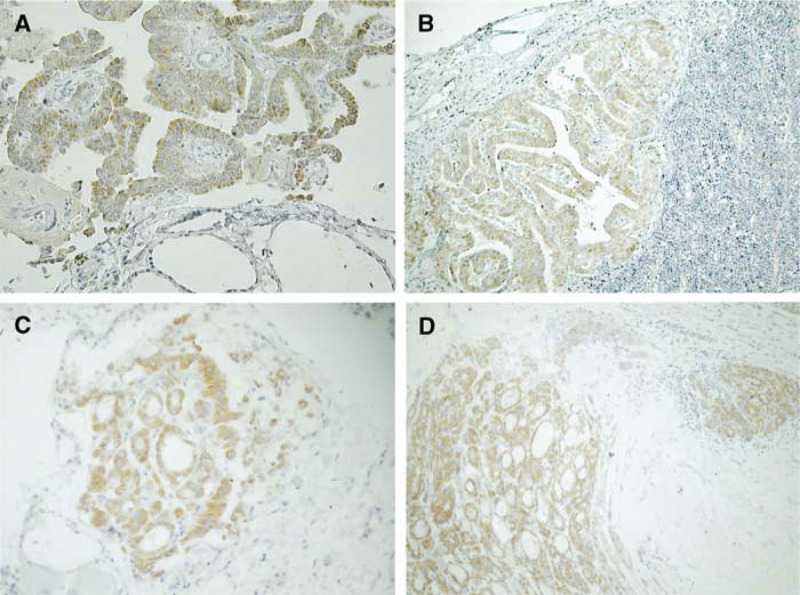
Immunohistochemical expression of the LAR P-subunit (YU1) in thyroid tumours. (**A**) papillary carcinoma, judged as (++), while the normal thyroid glands were negative (× 100), (**B**) papillary carcinoma, metastatic to lymph node (× 100), (**C**) follicular adenoma showing focally positive immunostaining, judged as (+) because positive tumour cells were 20–50% in the lesion (× 100), (**D**) follicular adenoma showing strong positive staining in invasive areas (diagnosed as invasive adenoma) (× 40).

Immunoreactivity was expressed as: (−) <20% of cells immunopositive; (+) 20–50% of cells immunopositive; (++) >50% of cells immunopositive.

summarises the results of YU1 immunostaining in thyroid tissues. As can be seen, papillary and follicular carcinomas stained more strongly and more frequently than any other tumour type (Figure 3A and B

). The limited number of anaplastic and medullary carcinomas were negative for YU1 as were the cases of nodular goitre, Basedow's disease and Hashimoto's thyroiditis. Of the 128 cases of histologically benign thyroid adenomas, only 22 (17.2%) stained positive. Fully half of these YU1-positive adenomas exhibited focal, as opposed to diffuse, immunoreactivity (Figure 3C); however, the other half of these cases (11 out of 22) showed invasive growth into the capsule, but had not spread into vessels or lymph channels (Figure 3D).

Figure 3A also demonstrates the very weak to nonexistent staining of normal follicular cells. The expression pattern of YU1 in papillary carcinoma was generally diffuse and intense in cytoplasm with only occasional nuclear staining. We also confirmed that what we saw was indeed expression of the LAR P-subunit in thyroid papillary carcinomas and in the corresponding normal tissues by Western blot analysis (Figure 1). YU1 was strongly expressed in 41.2% of tumours (87 of 211), and moderately positive in 54.5% of tumours (115 of 211); the remaining 4% of tumours (nine of 211) examined demonstrated little to no immunoreactivity. However, five of these nine immunonegative tumours were pretreated with Plank Rychlo solution or ethylenediamine tetraacetate (EDTA) for decalcification, which might have affected their ability to express YU1. However, EDTA treatment was not a factor for the three immunonegative follicular carcinomas. Fully 89.3% of follicular carcinomas (25 of 28) expressed YU1 to some extent, with 21% (six of 28) demonstrating strong expression and 67.9% (19 of 28) displaying moderate expression. Only 11% of these tumours were negative. The frequency of YU1 expression in papillary and follicular carcinomas was significantly different from that seen in both anaplastic and medullary carcinomas (*P*=0.017) and in benign diseases (*P*<0.001). There were no significant differences apparent between age or sex and immunohistochemical expression of YU1 (data not shown).

## DISCUSSION

In 1992, Streuli *et al* (1992) reported that the LAR E-subunit was shed from the surface of HeLa cells in culture. Subsequent investigations on LAR expression in mammalian tumours and cell lines, therefore, involved the use of antibodies raised against the LAR E-subunit (Li *et al*, 1996; Yang *et al*, 1999, 2000) with largely negative results in the tissues examined. Here we show that not only is the LAR P-subunit expressed weakly in a number of normal and tumourous human tissues but also that protein levels are markedly increased in thyroid carcinoma, particularly in malignant papillary type cancers. The strong LAR P-subunit expression seen in thyroid carcinoma at significantly high frequencies contrasts with the relatively lower expression frequencies exhibited by other types of tumours and parallels previous data on PTPase expression in tumours (Zhai *et al*, 1993; Wiener *et al*, 1994; Elson and Leder, 1995; Li *et al*, 1996; Norman *et al*, 1998; Yang *et al*, 1999, 2000). Further, our findings are consistent with theories linking changes in PTPase expression to thyroid tumorigenesis.

It is fairly clear that two kinds of function are carried out by PTPases acting in concert with receptor PTKs (Villa-Moruzzi *et al*, 1998). The first function lies in the positive regulation of signalling pathways; PTPases bind the phosphorylated receptor or adaptor, which then become functional towards their substrates. The Src homology 2 (SH2) domain-containing PTP, SHP2, thus has a positive effect on cellular signalling (Tonks and Neel, 1996). Overexpression of PTP *α* is also necessary for the dephosphorylation and activation of the Src kinase (Zheng *et al*, 1992). Abnormal expression of PTK genes, such as insulin-like growth factor receptor (IGF-IR), epidermal growth factor receptor (EGFR), focal adhesion kinase (FAK), or the proto-oncogenes RET and NyK/mer, has been shown to correlate with progression in a variety of tumours (Luttrell *et al*, 1994; Liu *et al*, 1995; Resnik *et al*, 1998). A number of tyrosine kinase genes are, in fact, expressed in thyroid, breast and prostate cancers (Weier *et al*, 2001). It is possible that our demonstrations of LAR overexpression in some other types of tissues and tumours may be because of its role in dephosphorylation of receptor PTKs.

The second function of PTPases is negative signal transduction. PTPases contribute to the return to a normal functional state by dephosphorylating the receptor PTK; this is a major function of the receptor-like PTPases. Overexpression of LAR downregulates insulin-stimulated cellular responses in hepatoma cells (Li *et al*, 1996), suggesting that LAR acts as a physiological modulator. Phosphorylation may also regulate tumour cell adhesion molecules. Loss of these molecules has been correlated with increased tumour invasiveness and change in phenotype (Bukholm *et al*, 1998). Such molecules, including p120, catenin, E-cadherin, *γ*-catenin (plakoglobin) and *β*-catenin, are apparently regulated by tyrosine phosphorylation (Kanner *et al*, 1991; Hinck *et al*, 1994; Xu and Carpenter, 1999). LAR expression was evidently re-gulated by contact inhibition via E-cadherin-dependent cell–cell communication in an *in vitro* study (Symons *et al*, 2002). Although we could not find any correlation between LAR expression and tumour differentiation in this study, expression may not be because of phosphorylation mediated in this particular fashion.

The *RET* gene codes for a transmembrane tyrosine kinase that displays a cadherin-like domain and a cysteine-rich motif in the extracellular region. Rearrangement of the RET tyrosine-kinase receptor with different genes, especially RET/PTC1 and PTC3 generated by the fusion of *RET* to the *H4* or *RFG* genes, respectively, is frequently found in thyroid papillary carcinoma, particularly in childhood radiation-induced thyroid cancers (Bongarzone *et al*, 1994; Santoro *et al*, 1994; Nikiforov *et al*, 1997; Jhiang, 2000). A recent study demonstrated that LAR overexpression decreased the oncogenic activity of a RET/MEN2A mutation when LAR and RET were cotransfected with a MEN2A or MEN2B mutation into an NIH3T3 cell line, possibly owing to interference with RET dimerisation (Qiao *et al*, 2001). The phenomenon was observed in both an *in vitro* colony formation assay as well as in an *in vivo* tumorigenicity assay using SCID mice. Interestingly, RET/PTC and the RET/MEN2A oncoproteins have constitutive kinase activity consequent to ligand-independent dimerisation (Monaco *et al*, 2001). We therefore suggest that LAR overexpression may affect RET/PTC dimerisation as well as kinase activity, and lead to reduced oncogenic potential, especially in papillary carcinoma.

A number of diagnostic markers have been reported in thyroid neoplasia. Recent interest has focused on the expression of galectin-3 and thyroid transcription factor-1 (TTF-1). Studies demonstrated the positivities of galectin-3 in 100% of papillary carcinomas, 90–100% of follicular carcinomas, 50–80% of medullary carcinomas, 0–33% of follicular adenomas and 0–38% of nodular goitres (Beesley *et al*, 2002). Thyroid transcription factor-1 also expressed 100% of differentiated follicular tumours including follicular adenoma, follicular carcinoma and papillary carcinoma, but only 25% of undifferentiated carcinoma of the thyroid (Katoh *et al*, 2000). These markers were often expressed in benign lesions as well as malignant lesions, being due to the different mechanisms from LAR P-subunit.

We have demonstrated that overexpression of LAR P-subunit specifically occurs in thyroid cancers, particularly in papillary and follicular carcinomas. In addition, of the 128 thyroid follicular adenomas examined, 22 tumours were positive for LAR P-subunit and half of these lesions demonstrated invasive growth into the capsule. The focal immunoreactive patterns of LAR expression in these adenomas suggest that YU1-positive cells have malignant potential and that adenomas expressing LAR P-subunit proteins have a greater propensity to progress to invasive and/or metastatic disease. Little information is available on the signal transducers involved in *RET* oncogene signalling. However, an increase in LAR may also represent a molecular mechanism for interfering with the kinase activity of RET/PTC and reducing its oncogenic activity. If so, monoclonal antibody YU1 against LAR P-subunit might be useful as both a therapeutic agent as well as a tumour marker. At the very least, it would seem to have a role in further analysis clarifying the role of PTPases in tumorigenesis.
